# Microwave Ablation Using Four-Tine Antenna: Effects of Blood Flow Velocity, Vessel Location, and Total Displacement on Porous Hepatic Cancer Tissue

**DOI:** 10.1155/2016/4846738

**Published:** 2016-08-24

**Authors:** Montree Chaichanyut, Supan Tungjitkusolmun

**Affiliations:** Department of Electronic Engineering, Faculty of Engineering, King Mongkut's Institute of Technology Ladkrabang, Bangkok 10520, Thailand

## Abstract

This research is concerned with microwave ablation analyses using a 2.45 GHz four-tine (4T) antenna for hepatic cancer tissue. In the study, three-dimensional finite-element models were utilized to examine the tissue temperature distributions during and after MW ablation. A preliminary study was first carried out with regard to the specific absorption rates along the 4T antenna insertion depths and the temperature distributions inside the solid and porous liver models with either 3 cm-in-diameter tumor or 5 cm-in-diameter tumor. Based on the preliminary results, the porous models were further examined for the effect of varying blood flow velocities (0–200 cm/s) with a 1 cm-in-diameter blood vessel next to the antenna and also for the effect of vessel-antenna locations (0, 0.8, and 1.3 cm) with a constant blood flow velocity of 16.7 cm/s. All scenarios were simulated under temperature-controlled mode (90°C). The findings revealed that the blood flow velocity and vessel location influence the ablation effectiveness and that increased blood flow inhibits heat transfer to the vessel wall. At the nearest and farthest vessel-antenna locations (0 and 1.3 cm), approximately 90.3% and 99.55% of the cancer cells were eradicated except for the areas adjacent to the vessel. In addition, total tissue thermal displacement is 5.9 mm which is 6.59% of the total length of the overall model.

## 1. Introduction 

Theoretically, microwave (MW) ablation possesses a number of advantages over the conventional radiofrequency (RF) ablation technique. In RF ablation (300 kHz–1 MHz), Joule heating is mainly generated by conduction current, while both conduction and displacement currents cause tissue temperatures to increase in MW ablation (generally at 915 MHz or 2.45 GHz) [1].

The conventional RF ablation method has proved effective in treatment of liver cancer with diameter of less than 3 cm [2, 3] but is afflicted with increased tissue impedance and the subsequent tissue boiling and charring [4]. In other words, the zone of active tissue treated with RF ablation is limited to a few millimeters surrounding an active electrode [5]. The majority of tissue heating is thus due to thermal conduction, which decreases exponentially away from the source. RF heating requires an electrical conduction path while MW is capable of propagating through material with low or zero conductivity (or high impedance) [6].

For cancer treatment with MW ablation, a thin antenna is inserted into a tumor through either open surgery or laparoscopy or percutaneously to deposit an electromagnetic (EM) field on the cancer tissue. Heating is induced using the conductive and displacement currents to affect the coagulative necrosis of tumor cells upon the temperatures reaching 50°C or above. The MW ablation zone characteristics (e.g., size and shape) are primarily subject to the antenna design [7–20]. Typically, an MW antenna is fed with a coaxial cable, whose topology and feed structure are optimized to achieve the intended heating pattern while choking the currents that flow on the outer surface of the outer conductor of the feeding coaxial cable. Examples of existing MW antennas are the monopole antenna [7–11], dipole or floating sleeve dipole antenna [7–9, 12, 13], choke or cap-choke antenna [7, 8, 12, 13], triaxial antenna [10, 16], metal tip monopole antenna [6], open tip monopole antenna [11, 17], and an open slot and multislot antennas [18–20]. These aforementioned antennas are most appropriate for teardrop-shaped tumors. In addition, tumors could develop near a blood vessel or cystic masses, thereby impeding the propagation of MW fields and thermal distribution.

**Figure 1:**
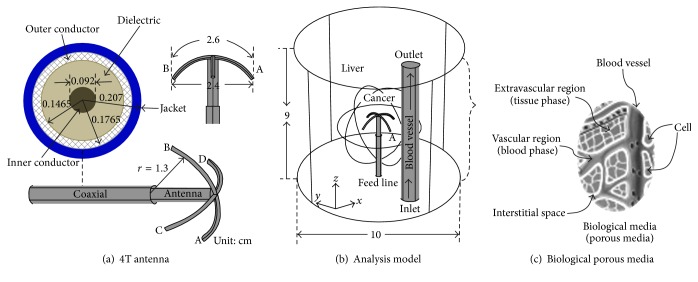
FEM model for liver ablation using the 4T antenna. The 4T antenna is fully deployed in the liver and a single 1 cm blood vessel is located 1.3 cm to the right of point A (four-tine (A, B, C, and D)).

In [21–24], the authors have documented the advantages of simultaneous activation of multiple antennas for achieving large ablative zones. The utilization of multiantenna systems requires determining the optimal antenna spacing for realization of the maximum ablative zone without indentations, since the presence of such indentations indicates incomplete destruction of the targeted tumor. To address the multiantenna's indentation issue, this current research thus presents a novel four-tine (4T) MW antenna that could potentially elevate tissue temperatures of large area without indentations (Figure 1(a)). In the antenna design, the inner conductor of a monopole antenna is connected to four conducting tines in an umbrella-like array arrangement similar to the four-tine RF probe in [2, 25, 26]. The 4T MW antenna is placed inside a penetrating sleeve prior to introducing into the cancerous liver tissue. Once the sleeve is properly lodged inside the tumor, the 4T array can be fully deployed as illustrated in Figures 1(a)-1(b).

Most previous researches on MW ablation for hepatic cancer regarded the liver tissue as a homogeneous solid mass and also focused exclusively on heat conduction within the tissue. Pennes's bioheat equation describes thermal conduction in the tissue and vascular system, blood perfusion, and metabolic heat generation [13–19].

Biological tissue comprises three components: blood vessels, cells, and interstitial space. In addition, biological media can be categorized as the vascular region (blood vessels) and extravascular region (cells and the interstitial space). Thus, the anatomical structure of the liver can be represented more accurately as a blood-saturated porous tissue [27–29], in which the vascular region is regarded as a blood phase and the extravascular region is regarded as a tissue phase (solid matrix), as illustrated in Figure 1(c). When the temperature of biological tissue rises, its shape is deformed due to thermal strain [30, 31]. Nevertheless, the majority of existing studies on MW ablation for hepatic cancer have focused predominantly on the EM wave propagation and heat transfer and also failed to explore the issue of mechanical tissue deformation.

This research has investigated the specific absorption rates (SARs) in the liver tissue using a 2.4 cm diameter 4T antenna (upon fully deployed). The temperature distributions of the solid and porous liver tissue models during and after MW ablation were examined and compared. In addition, the effects of blood flow velocity and vessel location on the temperature distributions in the liver tissue were determined. The total displacements of the porous liver tissue model during and after MW ablation were also investigated.

## 2. Materials and Methods 

### 2.1. Mathematical Descriptions of the Simulation Models

**Figure 2:**
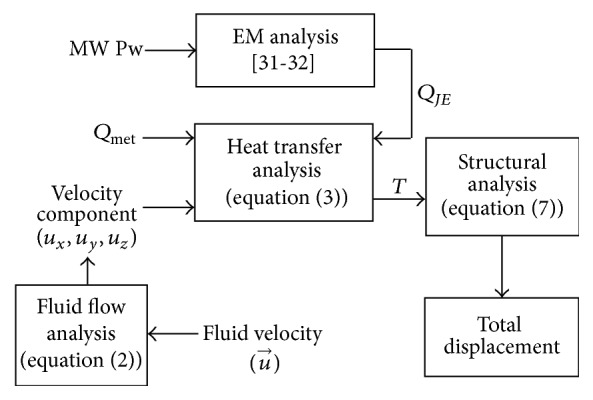
Block diagram of the mathematical analysis relevant to this research.

Figure 2 depicts a block diagram of the mathematical analysis pertinent to this research whose constituents encompass the EM, heat transfer, fluid flow, and mechanical changes analyses in MW ablation. The descriptions of each analysis are as follows.

*EM Wave Propagation Analysis*. The EM wave propagation in the liver tissue is rendered in a 3D model and calculated using Maxwell's equations. The general form of Maxwell's equations for transverse electromagnetic wave (TEM) mode is derived assuming a harmonic propagation and is simplified to demonstrate the EM field in a biological medium [32, 33].

*Fluid Flow Analysis*. This research utilized the incompressible Navier-Stokes (N-S) model to simulate the laminar flow conditions inside a blood vessel [34]. The general form of the N-S equation is valid for the flow inside the pores of a porous medium. Vafai and Tien [35] studied the heat transfer in porous media and proposed a modified momentum equation as follows:

(1) $$\begin{array}{c} \rho_{bl}\left(\;\langle \;\vec{u}\;\rangle \cdot \nabla \;\right)\langle \;\vec{u}\;\rangle =-\nabla \langle \;P\;\rangle +\mu_{bl}\nabla^{2}\langle \;\vec{u}\;\rangle -\frac{\mu_{bl}}{K}\phi \langle \;\vec{u}\;\rangle , \end{array}$$

where the subscript “bl” denotes blood and applies to an incompressible fluid (blood) of density *ρ*_bl_ (kg/m^3^), $\langle \vec{u}\rangle =(u_{x},u_{y},u_{z})$ is the average blood velocity (mm/s), 〈*P*〉 is the average fluid (blood) pressure (Pa), and *μ*_bl_ is the dynamic viscosity of blood (Pa·s). The dynamic viscosity of blood was 3.5 × 10^−3^ Pa·s; *K* and *ϕ* are, respectively, the permeability (m^2^) and the porosity of the porous liver tissue.

This research assumes a simple steady flow of incompressible fluids in which the inertia term $\rho_{bl}\left(\langle \vec{u}\rangle \cdot \nabla \right)\langle \vec{u}\rangle$ in (1) is neglected. The Navier-Stokes equation is thus reduced to the Brinkman equation given as follows [36]:

(2) $$\begin{array}{c} 0=-\nabla \langle \;P\;\rangle +\mu_{bl}\nabla^{2}\langle \;\vec{u}\;\rangle -\frac{\mu_{f}}{K}\phi \langle \;\vec{u}\;\rangle . \end{array}$$

*Heat Transfer Analysis*. The thermal distribution within the porous liver is obtained by solving the energy equations of tissue and blood phases, in which the absorbed MW power and an internal heat source (i.e., metabolic heat source) are included. In addition, this research assumes that the tissue and blood temperatures are identical (local thermal equilibrium (LTE)) [37].

According to [38], Pennes's bioheat equation fails to account for the effect of directional blood flow on heat transfer. To model the thermal energy propagation through the tissue and vessel from the ablation zone, this research utilized a convection-conduction Klinger model with the assumption of quasi-steady state as follows:

(3) ρcpts∂Tts∂t+ϕρcpblu→·∇T=∇·kts∇Tts+Qmet+QJE,

where the subscripts “ts” and “bl,” respectively, denote the tissue and blood phases. *T* is the temperature (°C); *ρ* is the density (kg/m^3^); *c*_*p*_ is the specific heat capacity (J/kg·°C);  *k* is the thermal conductivity (W/m·°C);  *ϕ* is the porosity which is the ratio of the blood volume to the total volume [27]. The metabolic heat generation rate of 33 800 W/m^3^ was used [39] and the external heat source was equal to the resistive heat generated by the EM field (*Q*_*JE*_ = *J* · *E* (W/m^3^)).

*Structural Analysis*. Heat transfer analysis provides us with the temperature at each element in the simulated model. A change in the temperature of a material contributes to variation in the material's overall size as a result of thermal strain [30, 31]. Temperature change could thus induce the dimension or shape variations in the liver tissue. In this research, it is assumed that the liver tissue is homogeneous and isotropic, and the strain (*ε*) consists of thermal (*ε*_th_), elastic (*ε*_el_), and initial (*ε*_0_) contributions such that

(4) $$\begin{array}{c} \varepsilon =\left(\;\varepsilon_{th}\;\right)+\left(\;\varepsilon_{el}\;\right)+\left(\;\varepsilon_{0}\;\right). \end{array}$$

To simplify the analysis and investigate the effect of thermal strain on the liver tissue, this research disregards the mechanical strain (*ε*_el_). The linear relationship between the thermal strain-induced deformation and temperature change is expressed as

(5) $$\begin{array}{c} \varepsilon_{th}=\alpha \left(\;T-T_{ref}\;\right), \end{array}$$

where *α* is the thermal expansion coefficient (1/°C) and *T*_ref_ is the reference temperature (°C).

Let us consider a small section (element) of the overall model as a small and finite cuboid in the Cartesian coordinates ({*x*, *y*, *z*}) and the dimensions of each side of the cuboid in initially undeformed state are, respectively, Δ*x*, Δ*y*, and Δ*z*. Due to the thermal strain, the cuboid is deformed, with displacement components in *x*, *y*, and *z* directions denoted by displacement vector *D*_*i*_ ≡ (*D*_*xi*_, *D*_*yi*_, *D*_*zi*_), where the subscript “*i*” is the index of the element. The deformed cuboid still remains a cuboid as shear strains are assumed to be zero throughout so that the angles are preserved. The dimensions of the cuboid sides nevertheless change, respectively, to Δ*x*_*i*_ + Δ*D*_*xi*_, Δ*y*_*i*_ + Δ*D*_*yi*_, and Δ*z*_*i*_ + Δ*D*_*zi*_, where Δ*D*_*xi*_, Δ*D*_*yi*_, and Δ*D*_*zi*_ denote the appropriate displacement increments.

Each component of the thermal strain depends on the displacements in each axis. Under the assumption of small displacements, the displacement vector (*D*_*xi*_, *D*_*yi*_, *D*_*zi*_) can be calculated from the thermal strain components (*ε*_*xxi*_, *ε*_*yyi*_, *ε*_*zzi*_) as follows [40]:

(6) εxxi=∂Dxi∂x;εyyi=∂Dyi∂y;εzzi=∂Dzi∂z.

Therefore, the total displacement (TD) of the model is

(7) $$\begin{array}{c} TD=\sqrt{\underset{i}{\sum }{\left(real\left(D_{i}\right)\right)}^{2}}. \end{array}$$

### 2.2. Properties of the Antenna

The four-tine array could be made from the shape-memory alloys (Ni-Ti alloy) with sharp edges and the 4T antenna was encapsulated in a guiding sleeve during insertion (Figures 1(a)-1(b)). Once the 4T antenna was placed at the target location, we pushed the 4T antenna forward into the liver to enable the four-tine array to penetrate into tissue. Once the four-tine array was fully deployed, the guiding sleeve was withdrawn from the liver.

### 2.3. Experimental Validation Setup

In order to validate the computational results from this study, we built a prototype 4T antenna based upon the results of the optimization and then tested this device in egg white and in swine liver. MW ablation was performed with input power of 50 W for a duration of 900 s. The power inputs were constantly varied to maintain the maximum temperature of 90°C. The thermocouple was placed 0.5 cm from the distal end of the antenna to measure temperature and was used as feedback to control power to maintain the maximum temperature of 90°C. Measurements were performed by inserting 4T antenna in 250 cm^3^ egg white and in 10 cm × 10 cm freshly excised swine liver obtained from a local slaughterhouse.

The prototype 4T antenna was constructed with the same dimensions as the model using a Multiflex 141 Coaxial Cable (Micro-Coax, Huber+ Suhner AG. RF Industrial, Herisau, Switzerland). The entire outer and the inner conductors are made from silver-plated copper wire. The coaxial dielectric used is a low-loss polytetrafuoroethylene (PTFE). Dimensions of the coaxial cable used to build the antenna assembly are provided in Table 1.

### 2.4. Analysis Assumptions

This research investigates the phenomenon within solid and porous biological media during and after MW ablation. With reference to Figure 1(c), in which the biological medium is porous, this research is carried out based upon the following assumptions:

- The outer surface of the porous liver is truncated by a scattering boundary condition (waves could pass through boundary without reflection) and the propagation of EM wave is confined to the liver tissue.
- The porous liver tissue is homogenous, thermally isotropic, and blood-saturated [41].
- Neither phase changes within the porous liver nor energy exchange through the outer surface of the porous liver takes place [42].
- The porosities and thermal properties of the porous liver are assumed to be constant [43].
- The initial temperature within the liver tissue is assumed to be uniform (37°C).

### 2.5. Comparison of Non-Blood Vessel Solid and Porous Models

In the finite-element (FE) analyses of the non-blood vessel models, the proposed 4T antenna was situated at the center of the liver. The antenna was first inserted from the tumor base along *z*-axis until the antenna tip was 0.7 cm above the center of the spherical tumor.  Figure 1(b) illustrates the FE analyses for both the solid and porous liver tissue models. The overall FE models are of cylindrical shape (diameter = 10 cm and length = 9 cm) with spherical hepatic cancer tissue of either 3 cm or 5 cm in diameter.

The boundary conditions and material properties were assigned prior to solving the thermal-electrical problems. In the MW ablation, the initial power was set at 50 W and the maximum temperature was monitored. Upon the hepatic cancer tissue temperature reaching 90°C, the power inputs were constantly varied to maintain the maximum temperature of 90°C. We limited the maximum temperature to 90°C to avoid situation where water in tissue reached boiling point which will cause bubbles. Similar temperature limit has also been used in other studies [24]. We selected the temperature-controlled mode, since it is more widely used in clinical practice to help reduce the incidence of overheating of the catheter-tissue interface and coagulation.

In addition, comparisons were made between the specific absorption rates (SARs) of the solid and porous models and between their temperature distributions.

### 2.6. Effects of Blood Flow Velocity

**Figure 3:**
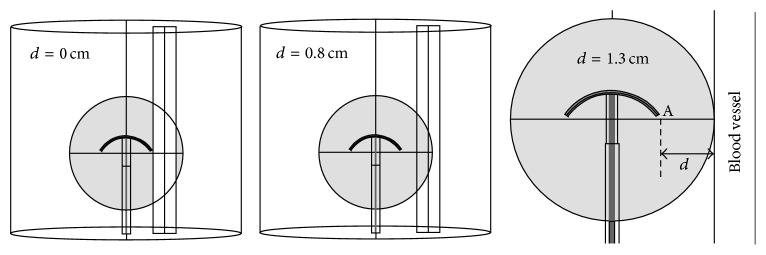
FEM model for liver ablation using the 4T MW antenna. The antenna is fully deployed in the liver and the antenna-vessel locations are 0, 0.8, and 1.3 cm to the right of point A (i.e., one of the distal ends of the 4T antenna).

In this research, an extreme case was simulated in which a 1 cm-in-diameter blood vessel was deliberately placed in immediate contact with point A and the hepatic cancer size was 5 cm in diameter (Figure 3). The blood flow velocity was varied between 0 cm/s and 200 cm/s to investigate the effect of blood flow on the temperature distribution and lesion formation during and after MW ablation. The variations in blood flow velocity range from 0 cm/s, 0.1 cm/s, 0.5 cm/s, 1 cm/s, 5 cm/s, 10 cm/s, 15 cm/s, 15.8 cm/s, 16.7 cm/s, 19.4 cm/s, 50 cm/s, 100 cm/s, and 200 cm/s.

### 2.7. Effect of Blood Vessel Location on Lesion Formation and Tissue Deformation

To determine the effect of blood vessel location on the temperature distribution, the distances of the 1 cm-in-diameter blood vessel from point A (i.e., one of the distal ends of the 4T antenna) in the 5 cm-in-diameter hepatic cancer porous model were varied between 0, 0.8, and 1.3 cm, as illustrated in Figure 3. The 3D FE analyses were thus performed for the following cases.

**Case 1 .**

The blood vessel was in immediate contact (0 cm) with point A and parallel to the 4T antenna.

**Case 2 .**

The blood vessel was 0.8 cm from point A and parallel to the 4T antenna. The blood vessel center was situated at the outer edge of the hepatic cancer.

**Case 3 .**

The blood vessel was 1.3 cm from point A and parallel to the 4T antenna. The blood vessel's outer wall was in contact with the hepatic cancer's outer edge.

Similar to Section 2.4 and Section 2.5, the boundary conditions and material properties were established prior to solving the thermal-electric-fluid-structural problems, except for the blood flow velocity which was assumed to be constant at 16.7 cm/s [44]. Comparisons were made with regard to the temperature distributions between the three distances to determine the effect of the location of blood vessel on the lesion formation.

### 2.8. Material Properties

Tables 1 and 2, respectively, tabulate dimensions and the properties of the proposed microwave antenna components, tissue and blood vessel, used in the FE models [15, 20, 25, 45–48]. All conductors of 4T antennas are assumed to be perfect electric conductors (PEC).

### 2.9. Meshing

A Cauchy convergence test was performed to determine appropriate mesh sizes for the simulated models and the threshold for maximum temperature difference was set at 0.1°C [49]. The mesh of all the liver tissue models was finer at the areas surrounding the four distal ends of the antenna (minimum grid size of 0.001 cm) and was coarser at locations farther away from the antenna tip (maximum grid size of 1 cm). The overall solid and porous FE models were of cylindrical shape (10 cm in diameter and 9 cm in length) with approximately 420 000 tetrahedral elements individually.

### 2.10. Software

Since the antenna geometry is complex, the numerical modeling was utilized to perform the 3D FE analyses to obtain the temperature distributions during and after MW ablation. The FE models were analyzed using COMSOL Multiphysics 5.1 (COMSOL, Inc., Burlington, MA) run on a PC with 3.60 GHz Intel® Pentium CORE*™* i7-3820 CPU, 24 GB of RAM, and 1 TB of hard disk space. The simulations performed with COMSOL Multiphysics included the RF module, heat transfer (bioheat equation) module, fluid flow module, and structure-mechanics module.

## 3. Research Results

Upon the establishment of the geometrical model, the FE mesh, and the material properties and boundary conditions, the FE analyses were carried out and postprocessing was performed for the simulation results as below.

### 3.1. Properties of the 4T Antenna

**Figure 4:**
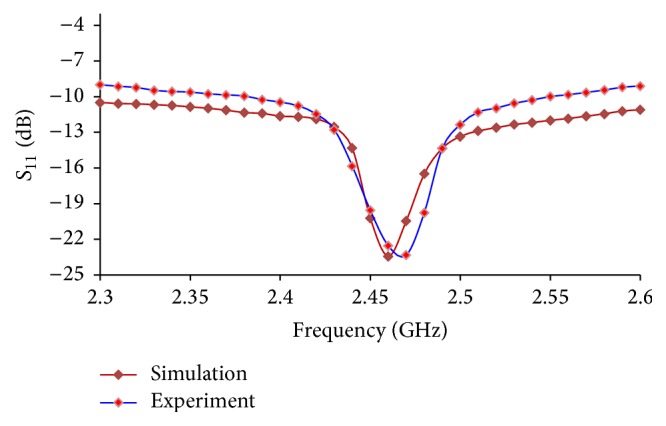
Frequency response of 4T antenna (*S*_11_).

We measured* S*_11_ of the 4T antenna in the range of 2.0 to 2.6 GHz in a 50 MHz step to characterize the frequency response of the antenna. We tested the fabricated prototype by measuring* S*_11_ using the Bird Site Analyzer Model SA-6000 EX (Bird Electronic Corporation, Cleveland, OH).  Figure 4 illustrates simulated* S*_11_ amplitude of 4T antenna versus those from experimental measurements.* S*_11_ at 2.45 GHz from the simulation result and experimental measurements were −20.21 dB and −19.56 dB, respectively. The discrepancies in* S*_11_ between FE simulations and the* in vitro* study was approximately 3.33%.

### 3.2. Results from* In Vitro* Experiments

**Figure 5:**
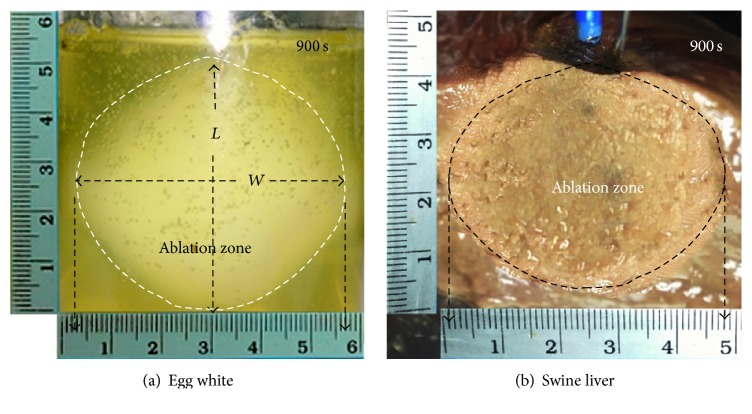
Ablation zones created in* in vitro* experiments at the end of a 900 s MWA procedure (egg white, 5.6 cm × 5.05 cm, and swine liver, 4.85 cm × 4.15 cm (size = *W* × *L*)).

Figures 5(a) and 5(b) show ablation zones after 900 s in egg white (5.6 cm × 5.05 cm) and in swine liver (4.85 cm × 4.15 cm), respectively. We compared the results from* in vitro* experiments and FE simulation of porous tissue model (no blood vessel) in Section 3.3.

### 3.3. Comparison of the Solid and Porous Models

**Figure 6:**
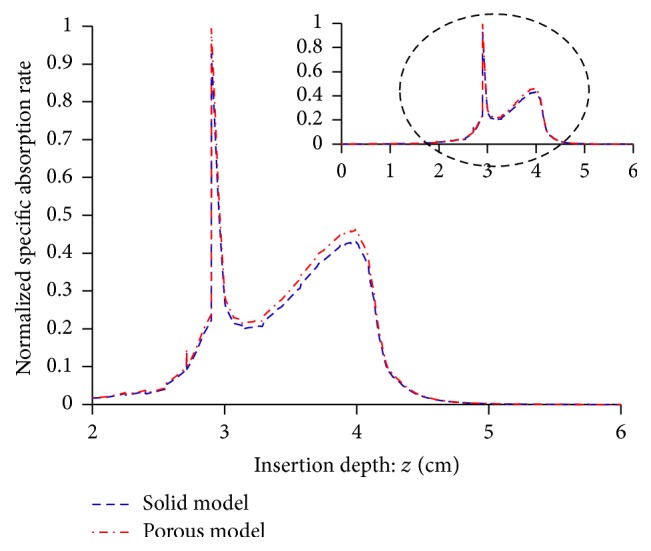
SAR along the insertion depths of 4T antenna.

*SAR Distributions*.  Figure 6 illustrates the specific absorption rates relative to the antenna insertion depths (*z* = 0–0.6 cm) from the FE analyses of the solid and porous liver tissue models, respectively. The SARs along *z*-axis at 0.125 cm radius were normalized. The local peaks of the SAR curves for both solid and porous models occurred at the interface between the distal end of the monopole antenna and the four tines (i.e., points A, B, C, and D), as seen in Figure 1(a). The SAR profile curves of the solid and porous models exhibited similar trends with slightly different amplitudes.

**Figure 7:**
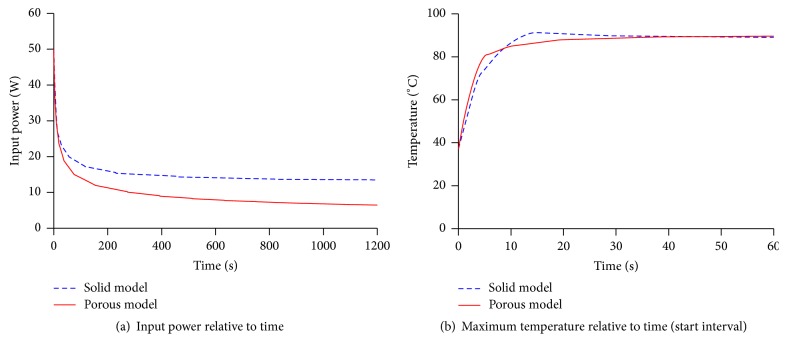
Microwave power delivery and maximum temperatures relative to time.

*Temperature and Power Observations*. Figures 7(a)-7(b), respectively, depict the MW power delivery and the resulting maximum temperatures relative to time from the FE analyses of the solid and porous liver tissue models. The MW power delivery was varied to maintain the target temperature of 90 ± 1°C. Initially, the tissue temperature rose rapidly to 90°C in 12 s and 35 s, respectively, for the solid and porous models with a 50 W power delivery which was subsequently adjusted for maintenance of the target temperature. The average power uses for the solid and porous models were 15 W and 8 W, respectively (Figure 7).

**Figure 8:**
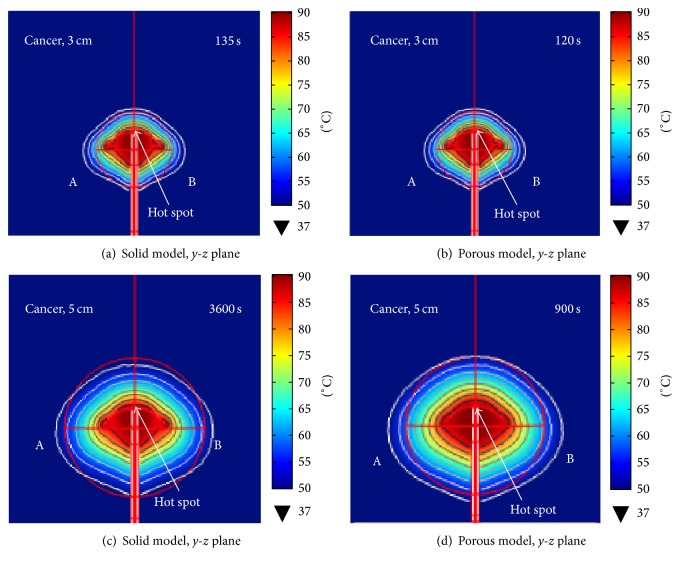
The cross section (in the* y*-*z* plane) of temperature distribution for 3 cm-in-diameter tumor (a) in solid model after 135 s and (b) in porous model after 120 s and for 5 cm-in-diameter tumor (c) in solid model after 3600 s and (d) in porous model after 900 s.

*Temperature Distributions*. The temperature-controlled (90°C) FE analyses were performed on both 3 cm-in-diameter and 5 cm-in-diameter hepatic tumors under the solid and porous liver tissue conditions. Figures 8(a)-8(b), respectively, illustrate the degrees of tissue destruction for the solid and porous models with 3 cm-in-diameter hepatic tumor. Figures 8(c)-8(d), respectively, demonstrate the extents of tissue destruction for the solid and porous models in the case of 5 cm-in-diameter hepatic tumor. In Figures 8(a)–8(d), the temperature distributions are symmetric around *z*-axis. The areas with maximum temperatures (“hot zones”) are those around the 4T antenna opening (Figure 8). The lesion formation for all cases was of* oculiform* shape.

The ablation durations for complete destruction (100%) of 3 cm-in-diameter hepatic tumor (volume = 14.14 cm^3^) for the solid and porous models were, respectively, 135 s and 120 s. In case of the 5 cm-in-diameter hepatic tumor, the ablation duration for complete destruction in the porous model was 900 s, while the maximum destruction in the solid model was a mere 93.60% even after 3600 s. In addition, the 4T antenna caused damage to the normal liver tissue in the vicinity of the hepatic tumor (Figures 8(c)-8(d)). The volumes of normal tissue destruction for the liver with 5 cm-in-diameter hepatic tumor were 8.19 cm^3^ and 33.56 cm^3^ for the solid and porous models, respectively.

The dimensions of ablation zone from FE simulations of porous model (6.23 cm × 5.28 cm) are slightly larger than those of the* in vitro* experiments but, otherwise, exhibit similar characteristics (*oculiform* shape). Thus, we conclude that our FE analyses offer satisfying result in this study as FE model was able to predict coagulation zone characteristics of 4T antenna MW ablation.

### 3.4. Effect of Blood Flow Velocity

**Figure 9:**
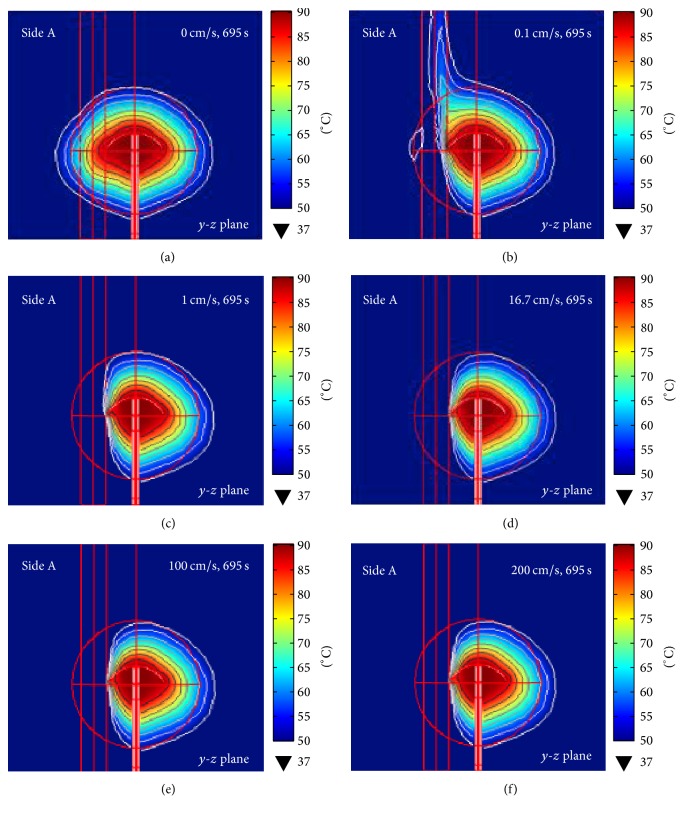
The cross sections (in the* y*-*z* plane) of temperature distribution in the porous model with a 10 mm-in-diameter blood vessel in immediate contact with point A and parallel to the 4T antenna (Figure 1(b)). The variations in the blood flow velocity range from (a) 0 cm/s, (b) 0.1 cm/s, (c) 1 cm/s, (d) 16.7 cm/s, (e) 100 cm/s, and (f) 200 cm/s.

*Temperature Distribution*. A preliminary MW ablation analysis was performed to identify the baseline duration for complete destruction of 5 cm hepatic cancer in the porous model with the 0 cm/s blood flow velocity and the result was 695 s.  Figure 9 illustrates the temperature distributions on the* y*-*z* plane of the porous model with varying blood flow velocities. The temperature distributions are asymmetric around the 4T antenna with the temperature distribution on side A extending upward toward the top due to the upstream flow (Figure 9(b)). In the case of blood flow velocities below 5 cm/s, the temperatures along the vessel wall were above 37°C. When the blood flow velocity was beyond 5 cm/s, the temperatures along the vessel wall remained at 37°C due to the high heat convection from blood flow and thereby more asymmetric temperature distributions. The high blood flow velocity resulted in smaller lesion dimensions as the lesion volume reduced from 62.87 cm^3^ (0 cm/s) to 56.63 cm^3^ (200 cm/s), as presented in Table 3. In the presence of blood flow velocity, the ablation of hepatic tumor was below 100% (i.e., complete destruction) as the hepatic tissues surrounding the blood vessel remained unablated (Figures 9(b)–9(f)).

### 3.5. Effect of Blood Vessel Location on Lesion Formation and Tissue Deformation

The effects of varying blood vessel (1 cm in diameter) locations (i.e., 0, 0.8, and 1.3 from point A) on the lesion formation in the 5 cm hepatic cancer porous model at the constant 16.7 cm/s flow velocity were determined by comparing the temperature distributions. In addition, the effects of thermal strain on tissue deformation were investigated.

**Figure 10:**
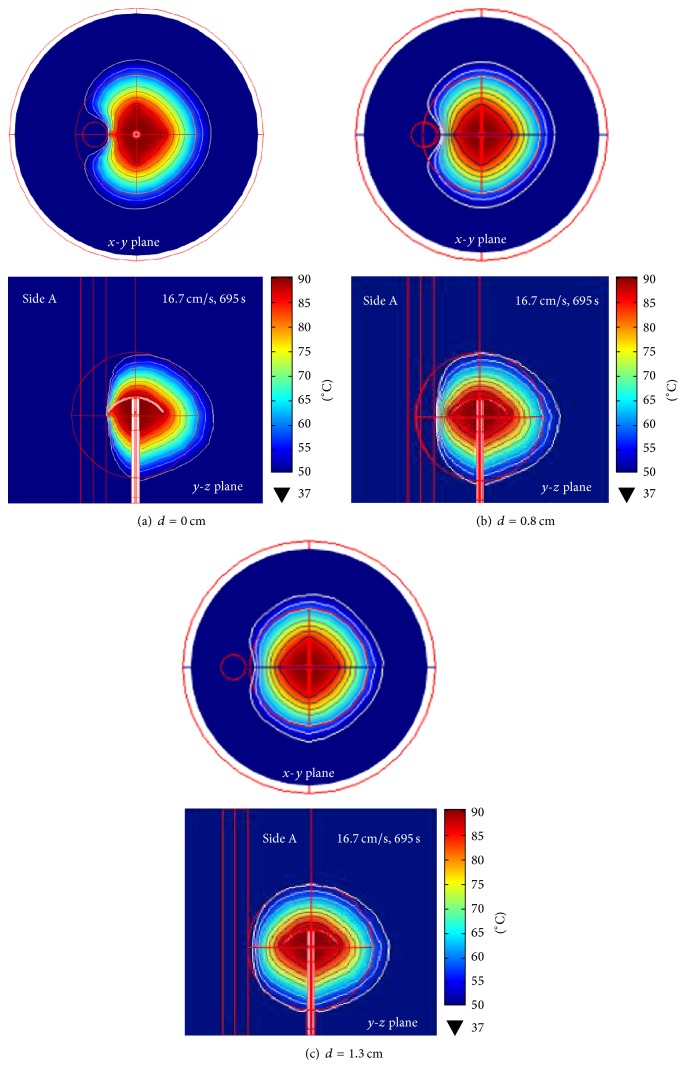
The cross sections (in the* x*-*y* plane and* y*-*z* plane) of temperature distribution in the 5 cm-in-tumor porous model: (a) 1 cm-in-diameter blood vessel in immediate contact with point A and parallel to the 4T antenna, (b) the blood vessel located 0.8 cm from point A and parallel to the antenna, and (c) the blood vessel located 1.3 cm from point A and parallel to the antenna.

*Temperature Distribution*.  Figure 10 illustrates the simulated temperature distributions on the* x*-*y* and* y*-*z* planes. Figures 10(a)–10(c), respectively, illustrate the temperature distributions for the blood vessel locations at 0, 0.8, and 1.3 cm. Contrary to the no blood vessel condition in which the lesion formation was symmetric, the lesion formations in the presence of blood vessel were asymmetric.

As presented in Table 3, the 4T antenna can induce the MW power within the cancerous tissue to destroy the hepatic cancer. At the 0 cm blood vessel location, approximately 90.30% of the hepatic cancer was ablated. At the 0.8 and 1.3 cm locations, the destruction rates were, respectively, 96.27% and 99.55%.

**Figure 11:**
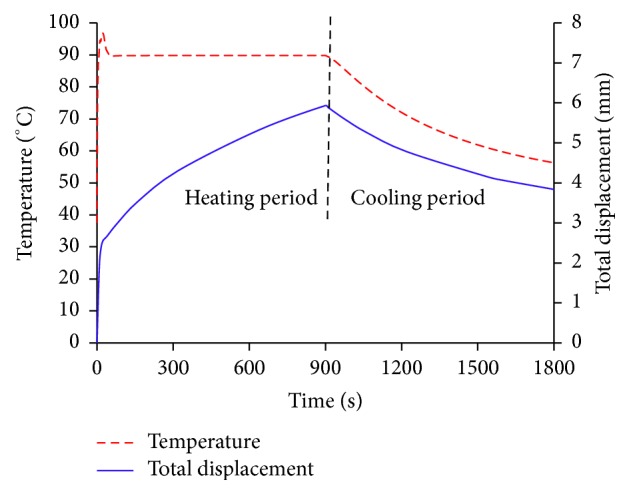
The depiction of the temperature and total displacement curve in the liver tissue due to thermal strain relative to time. The liver tissue is porous with 5 cm tumor with 0 cm vessel location and 16.7 cm/s blood flow velocity.

*Total Displacement*.  Figure 11 shows the simulated maximum temperatures and total displacements of the liver tissue due to thermal strain relative to time in case of the 5 cm tumor porous model with 0 cm vessel location and 16.7 cm/s blood flow velocity. The heating period of MW ablation and the subsequent cooling period were both 900 s. The total displacements increased sharply immediately after the thermal treatment commenced. During the heating period, tissue displacements continuously increased and decreased due to tissue necrosis (stiffening) during the cooling period. The tissue displacement reached a peak of 5.9 mm at 90°C. At the end of the cooling period, the temperature and displacements decreased to 56.3°C from originally 90°C (at termination of the heating period) and to 3.8 mm from 5.9 mm.

## 4. Discussions

The aim of this research is to construct a most realistic FE model that represents the tissue transformation during and after MW liver ablation. In the FE analyses, the 2.45 GHz 4T antenna was deployed in ablating hepatic cancer in the porous liver tissue model and then the tissue temperature distributions were determined.

The effects of varying blood flow velocities on temperature distribution and tissue deformation (via total displacement) given that the blood vessel was in immediate contact (0 cm) with the 4T antenna were determined. In addition, this research examined the impacts of varying blood vessel locations on the temperature distributions and tissue deformations.

The analyses of thermal destruction in the solid and porous models indicated different specific absorption rates (SAR) of the liver tissue during MW ablation. In the solid model analysis, the liver tissue was assumed to be homogeneous with no voids and in determination of the temperature distributions, only the EM properties, tissue thermal properties, and bioheat equation were used.

The porous liver tissue model was also assumed to be homogenous but contained small voids inside. The porous tissue is composed of tissue phase and interstitial space [27]. In the porous model analysis, in addition to the EM properties, tissue thermal properties, and bioheat equation, the tissue mechanical properties (i.e., porosity, viscosity, and Young's modulus, thermal expansion coefficient, and Poisson's ratio) are required to perform the thermal, electric, fluid flow, and structural analyses. By comparison, the simulated SAR and temperature were higher in the porous model than in the solid tissue, a phenomenon attributable to the higher electrical and thermal conductivities of fluid (i.e., blood) inside the porous tissue in relation to those of the tissue.

In the presence of the 1 cm blood vessel with high blood flow velocity, the temperature distribution pattern is asymmetric due to the loss of heat attributable to the blood flow, as seen in Figures 9-10 [25]. The flowing blood acts as a heat sink and thereby dissipates the thermal heat from the surrounding tissue. The percentage of hepatic tumor destruction and the shape of lesion formation are subject to the distance between the blood vessel and the 4T antenna. The high blood flow velocity contributes to the convective cooling effect and thus a lower tissue temperature in the areas adjacent to the blood vessel.

According to [50], the thermal displacement initially increases rapidly. If the heating was sustained, the displacement would continue to increase but at a slower pace. It could be concluded that the change in the tissue displacement characteristics is indicative of the heat-induced structural changes in the hepatic tissue. Furthermore, the thermal displacement decreases over the course of cooling but does not return to the preheating state.

Major limitations of analyses in this study are simplified assumptions (e.g., simple steady flow of incompressible fluids) in the blood flow and energy equations (see (1)-(2)). In addition, we assumed that the conductor of the 4T antenna was a perfect electric conductor. No phase change occurred within the liver tissue and no energy exchange through the outer surface of liver tissue was allowed. Future studies will address these limitations which will result in more accurate FE models.

## 5. Conclusion

This research has presented the 3D FE analyses of MW liver ablation using the 4T antenna under various conditions. The FE analyses were first carried out with the solid and porous liver models with either 3 cm-in-diameter tumor or 5 cm-in-diameter tumor to investigate the specific absorption rates (SARs) along the 4T antenna insertion depths and the temperature distributions. The porous liver tissue model with 5 cm tumor MW-ablated using the proposed 4T antenna was further examined with the introduction of the 1 cm-in-diameter blood vessel and variations of blood flow velocity (0–200 cm/s) and blood vessel location (0, 0.8, and 1.3 cm from a distal end of the 4T antenna). In addition, the mechanical tissue deformation and liver tissue displacement were also observed. This model-based approach is economically sensible and medically safe as a first step for the development of simple tools for improved therapeutic effect. To verify the FE analysis results, more realistic* in vitro *and* in vivo* experiments will be part of our future research.

## Figures and Tables

**Table 1:** Dimensions of model components.

Components	Dimensions	
	Length (cm)	Diameter (cm)
Inner conductor	4.2	0.092
Dielectric	4.2	0.293
Outer conductor	2.9	0.353
Antenna, four-tine antenna (A, B, C, and D)	1.3	0.092
Blood vessel	9.0	1.0
Tissue (cancer)	—	5.0
Tissue (liver)	9.0	10.0

**Table 2:** Material properties in the FE model [15, 20, 25, 41, 45–48].

Material	Electromagnetic		Thermal					Fluid		Mechanical		
	*ε*_*r*_	*σ*	*k*		*c*_*p*_		*ρ*	*ϕ*	*K*	*υ* ^*∗*^	*E*	*α*
			20–70°C	80–100°C	20–70°C	80–100°C						
	—	[S/m]	[W/m·°C]		[J/kg·°C]		[kg/m^3^]	—	m^2^	—	[Pa]	(1/°C)
PTFE	1.8 [15]	1.6 × 10^−5^ [15]	0.24 [15]		1050 [15]		1200 [15]	—	—	—	—	—
Blood vessel	58.30 [41]	2.54 [46]	0.543 [25]		4180 [25]		1000 [41]	1 [46]	—	0.49 [47]	2.2 × 10^5^ [47]	1 × 10^−4^ [48]

Tissue (cancer)	48.16 [41]	2.09 [42]	0.570 [41]	0.867 [20]	3960 [41]	3960 [41]	1040 [41]	0.7 [41]	2.72 × 10^−10^ [41]	0.48 [48]	10.2 × 10^6^ [48]	1 × 10^−4^ [48]
Tissue (liver)	43.03 [20]	1.69 [20, 45]	0.497 [41]	0.867 [20]	3600 [20, 45]	3858 [41]	1030 [46]	0.6 [41]	7.71 × 10^−11^ [41]	0.48 [48]	10.2 × 10^6^ [48]	1 × 10^−4^ [48]

^*∗*^ *υ* is Poisson's ratio.

**Table 3:** The resulting ablation volume of all cases.

Material model	Position^*∗*^ (cm)	Velocity (cm/s)	Time (s)	Ablation result of cancer of 5 cm diameter					
				Total liver volume (cm^3^)	Total cancer volume (cm^3^)	Total ablation (cm^3^)	Ablated liver volume (cm^3^)	Ablated cancer volume (cm^3^)	% Ablated cancer
Solid	No blood		900	641.66	65.48	59.38	2.78	56.60	86.44
			3600			69.48	8.19	61.29	93.60

Porous	No blood		900	641.66	65.48	99.04	33.56	65.48	100.00

Porous	0	0	695	641.66	62.87	96.51	33.64	62.87	100.00
		0.1				90.11	30.17	59.94	95.34
		0.5				83.36	23.74	59.61	94.82
		1				81.29	22.55	58.74	93.43
		5				78.96	21.85	57.11	90.84
		10				78.60	21.75	56.85	90.42
		15				78.62	21.80	56.82	90.38
		16.7				78.59	21.82	56.77	90.30
		19.4				78.49	21.76	56.73	90.23
		50				78.43	21.77	56.66	90.12
		100				78.40	21.75	56.65	90.10
		200				78.37	21.74	56.63	90.07

Porous	0	16.7	695	641.66	62.87	78.59	21.82	56.77	90.30
	0.8				64.29	85.66	23.77	61.90	96.27
	1.3				65.48	91.63	26.45	65.19	99.55

^*∗*^The distance of the blood vessel from point A of the 4T antenna.

## References

[1] Simon C. J., Dupuy D. E., Mayo-Smith W. W. (2005). Microwave ablation: principles and applications. *RadioGraphics*.

[2] Wood T. F., Rose D. M., Chung M., Allegra D. P., Foshag L. J., Bilchik A. J. (2000). Radiofrequency ablation of 231 unresectable hepatic tumors: indications, limitations, and complications. *Annals of Surgical Oncology*.

[3] Seror O., N'Kontchou G., Ibraheem M. (2008). Large (≥5.0-cm) HCCs: multipolar RF ablation with three internally cooled bipolar electrodes—initial experience in 26 patients. *Radiology*.

[4] Goldberg S. N., Gazelle G. S., Solbiati L., Rittman W. J., Mueller P. R. (1996). Radiofrequency tissue ablation: increased lesion diameter with a perfusion electrode. *Academic Radiology*.

[5] Organ L. W. (1976). Electrophysiologic principles of radio frequency lesion making. *Applied Neurophysiology*.

[6] Brace C. L. (2009). Radiofrequency and microwave ablation of the liver, lung, kidney, and bone: what are the differences?. *Current Problems in Diagnostic Radiology*.

[7] Prakash P., Converse M. C., Webster J. G., Mahvi D. M. Design optimization of coaxial antennas for hepatic microwave ablation using genetic algorithms.

[8] Chang Y., Che W., Yang L., Yang L., Chen G. Experimental studies on microwave ablation in vitro animal tissues with microwave percutaneous coagulator.

[9] Cepeda M. F. J., Vera A., Leija L. Electromagnetic hyperthermia ablation devices for breast cancer: state of the art and challenges for the future.

[10] Cavagnaro M., Tuzio A. G., Pisa S. The matching of microwave ablation antennas through a semi-analytic technique.

[11] Wang P., Brace C. L., Converse M. C., Webster J. G. (2009). Tumor boundary estimation through time-domain peaks monitoring: numerical predictions and experimental results in tissue-mimicking phantoms. *IEEE Transactions on Biomedical Engineering*.

[12] Wang P., Brace C. L. (2012). Tissue dielectric measurement using an interstitial dipole antenna. *IEEE Transactions on Biomedical Engineering*.

[13] Prakash P., Converse M. C., Webster J. G., Mahvi D. M. (2009). An optimal sliding choke antenna for hepatic microwave ablation. *IEEE Transactions on Biomedical Engineering*.

[14] Maini S., Marwaha A. Comparison of coaxial choke and extended tip choke antenna for interstitial microwave ablation of HCC.

[15] Brace C. L., Laeseke P. F., Van Der Weide D. W., Lee F. T. (2005). Microwave ablation with a triaxial antenna: results in ex vivo Bovine liver. *IEEE Transactions on Microwave Theory and Techniques*.

[16] Cavagnaro M., Amabile C., Bernardi P., Pisa S., Tosoratti N. (2011). A minimally invasive antenna for microwave ablation therapies: design, performances, and experimental assessment. *IEEE Transactions on Biomedical Engineering*.

[17] Kaur1 S., Maini S. (2014). Microwave Ablation therapy for the treatment of hepatocellular carcinoma using double slot interstitial antenna. *International Journal of Research in Computer Applications and Robotics*.

[18] Karampatzakis A., Tsanidis G., Kuhn S., Neufeld E., Kuster N., Samaras T. Computational study of the performance of single applicators and antenna arrays used in liver microwave ablation.

[19] Ortega-Palacios R., Vera A., Leija L. Microwave ablation coaxial antenna computational model slot antenna comparison.

[20] Phasukkit P., Tungjitkusolmun S., Sanpanich A. Finite element analysis on phase shift effect of multi-antenna array alignment for microwave liver ablation.

[21] Simon C. J., Dupuy D. E., Iannitti D. A. (2006). Intraoperative triple antenna hepatic microwave ablation. *American Journal of Roentgenology*.

[22] Wright A. S., Lee F. T., Mahvi D. M. (2003). Hepatic microwave ablation with multiple antennae results in synergistically larger zones of coagulation necrosis. *Annals of Surgical Oncology*.

[23] Wright A. S., Sampson L. A., Warner T. F., Mahvi D. M., Lee F. T. (2005). Radiofrequency versus microwave ablation in a hepatic porcine model. *Radiology*.

[24] Hines-Peralta A. U., Pirani N., Clegg P. (2006). Microwave ablation: results with a 2.45-GHz applicator in *ex vivo* bovine and *in vivo* porcine liver. *Radiology*.

[25] Tungjitkusolmun S., Staelin S. T., Haemmerich D. (2002). Three-dimensional finite-element analyses for radio-frequency hepatic tumor ablation. *IEEE Transactions on Biomedical Engineering*.

[26] Bilchik A. J., Wood T. F., Allegra D. P. (2001). Radiofrequency ablation of unresectable hepatic malignancies: lessons learned. *The Oncologist*.

[27] Nakayama A., Kuwahara F. (2008). A general bioheat transfer model based on the theory of porous media. *International Journal of Heat and Mass Transfer*.

[28] Pennes H. H. (1948). Analysis of tissue and arterial blood temperatures in the resting human forearm. *Journal of applied physiology*.

[29] Khaled A.-R. A., Vafai K. (2003). The role of porous media in modeling flow and heat transfer in biological tissues. *International Journal of Heat and Mass Transfer*.

[30] Shen W., Zhang J., Yang F. (2005). Modeling and numerical simulation of bioheat transfer and biomechanics in soft tissue. *Mathematical and Computer Modelling*.

[31] Xu F., Wen T., Lu T. J., Seffen K. A. (2008). Skin biothermomechanics for medical treatments. *Journal of the Mechanical Behavior of Biomedical Materials*.

[32] Cheng D. K. (1991). *Field and Wave Electromagnetics*.

[33] Jin J. (2002). *The Finite Element Method in Electromagnetics*.

[34] Keeet R. J., Coltrin M. E., Glarborg P. (2003). *Chemically Reacting Flow: Theory and Practice*.

[35] Vafai K., Tien C. L. (1981). Boundary and inertia effects on flow and heat transfer in porous media. *International Journal of Heat and Mass Transfer*.

[36] Batchelor G. K. (1967). *An Introduction to Fluid Dynamics*.

[37] Zhang Y. (2009). Generalized dual-phase lag bioheat equations based on nonequilibrium heat transfer in living biological tissues. *International Journal of Heat and Mass Transfer*.

[38] Peng T., O'Neill D. P., Payne S. J. (2011). A two-equation coupled system for determination of liver tissue temperature during thermal ablation. *International Journal of Heat and Mass Transfer*.

[39] Rabin Y., Shitzer A. (1998). Numerical solution of the multidimensional freezing problem during cryosurgery. *Journal of Biomechanical Engineering*.

[40] Roylance D. (2008). *Mechanical Properties of Materials*.

[41] Rattanadecho P., Keangin P. (2013). Numerical study of heat transfer and blood flow in two-layered porous liver tissue during microwave ablation process using single and double slot antenna. *International Journal of Heat and Mass Transfer*.

[42] Whitaker S. (1969). Fluid motion in porous media. *Industrial & Engineering Chemistry*.

[43] Slattery J. C. (1969). Single-phase flow through porous media. *AIChE Journal*.

[44] Zoli M., Magalotti D., Bianchi G. (1999). Total and functional hepatic blood flow decrease in parallel with ageing. *Age and Ageing*.

[45] Yang D., Converse M. C., Mahvi D. M., Webster J. G. (2007). Expanding the bioheat equation to include tissue internal water evaporation during heating. *IEEE Transactions on Biomedical Engineering*.

[46] Keangin P., Vafai K., Rattanadecho P. (2013). Electromagnetic field effects on biological materials. *International Journal of Heat and Mass Transfer*.

[47] Daniels M. (2008). *Temperature estimation with ultrasound [Ph.D. dissertation]*.

[48] Keangin P., Wessapan T., Rattanadecho P. (2011). Analysis of heat transfer in deformed liver cancer modeling treated using a microwave coaxial antenna. *Applied Thermal Engineering*.

[49] Tungjitkusolmun S., Woo E. J., Cao H., Tsai J. Z., Vorperian V. R., Webster J. G. (2000). Thermal-electrical finite element modelling for radio frequency cardiac ablation: effects of changes in myocardial properties. *Medical and Biological Engineering and Computing*.

[50] Maleke C., Konofagou E. E. (2008). Harmonic motion imaging for focused ultrasound (HMIFU): a fully integrated technique for sonication and monitoring of thermal ablation in tissues. *Physics in Medicine and Biology*.

